# Intermediate bronchial fistula caused by mediastinal drainage tube compression and fungal infection: a case report

**DOI:** 10.1186/s13019-019-1020-x

**Published:** 2019-11-07

**Authors:** Yahua Li, Kewei Ren, Liqun Ye, Jianzhuang Ren, Xinwei Han

**Affiliations:** 1 Department of Interventional Radiology, The First Affiliated Hospital of Zhengzhou University, Zhengzhou, 450052 People’s Republic of China; 2 Department of Respiratory Medicine, The First Affiliated Hospital of Zhengzhou University, Zhengzhou, 450052 People’s Republic of China

**Keywords:** Tracheal stent, Intermediate bronchial fistula, Fungal infection

## Abstract

**Background:**

Intermediate bronchial fistula formation caused by mediastinal drainage tube compression and fungal infection is rare.

**Case presentation:**

A 50-year-old male patient with type 2 diabetes was observed air filling in mediastinal drainage tube, 12 days after esophagectomy for esophageal squamous carcinoma. Based on the results of computed tomography, bronchoscopy and pathology, the diagnosis of intermediate bronchial fistula caused by mediastinal drainage tube compression and fungal infection was made. Anti-fungal drug and temporary covered metallic stent was used. After stent removed, the fistula was healed with some granulation hyperplasia. He was free from respiratory symptom during 1 year follow-up.

**Conclusion:**

Intermediate bronchial fistula caused by the combination of mediastinal drainage tube compression and fungal infection is rare. Timely stenting could boost the healing of fistula via granulation tissue proliferation.

## Background

Intermediate bronchial fistula formation caused by mediastinal drainage tube compression and fungal infection is rare. Stent placement as a minimally invasive therapeutic option may be recommend in such cases. Usually, silicon stent placement is the first choice. However, placement of small diameter silicon stent in small- to medium-caliber airway is not available in some regions. Here, we introduce the experience of small diameter individualized Y-shaped covered self-expandable metallic stent (SEMS) for intermediate bronchial fistula.

## Case presentation

**Fig. 1:**
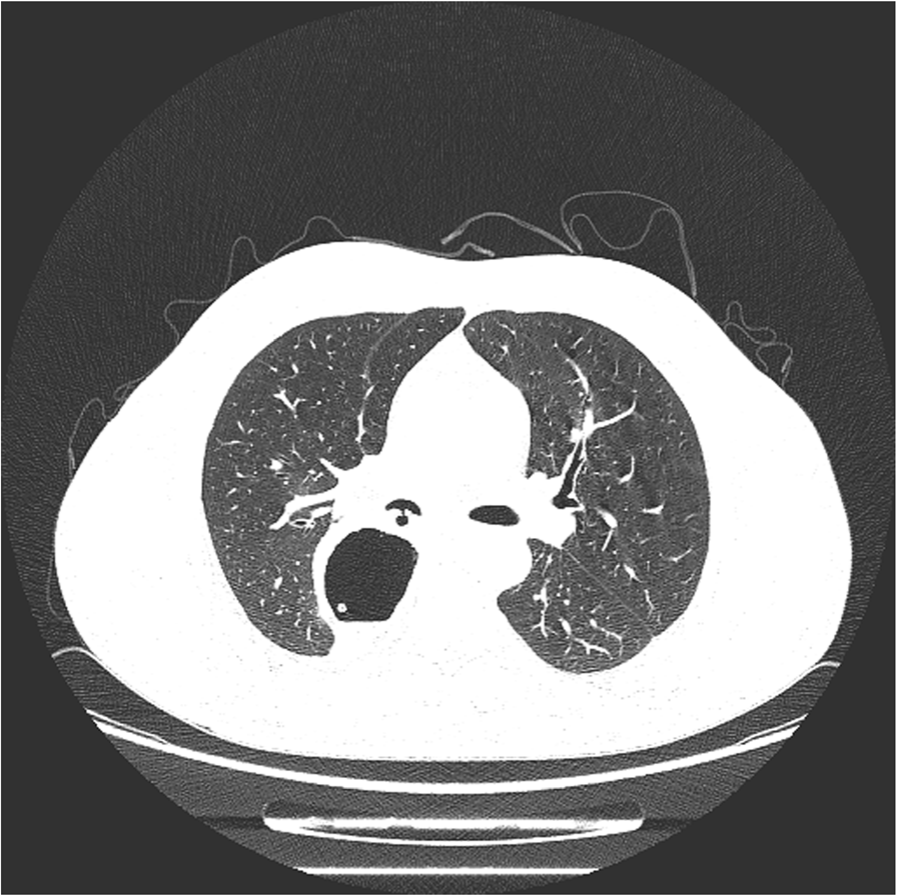
A connection between intermediate bronchus and mediastinal drainage tube tract was observed through lung window

**Fig. 2:**
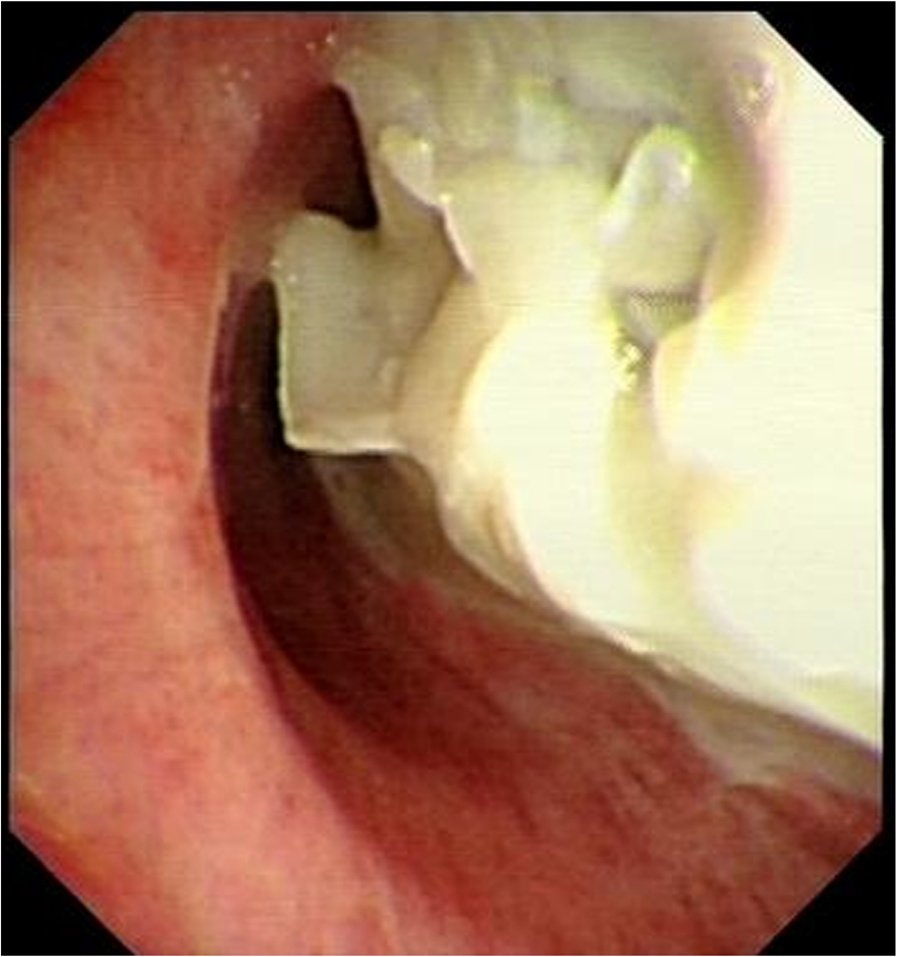
Bronchoscopy revealed that massive white necrotic materials attached the wall of intermediate bronchus

**Fig. 3:**
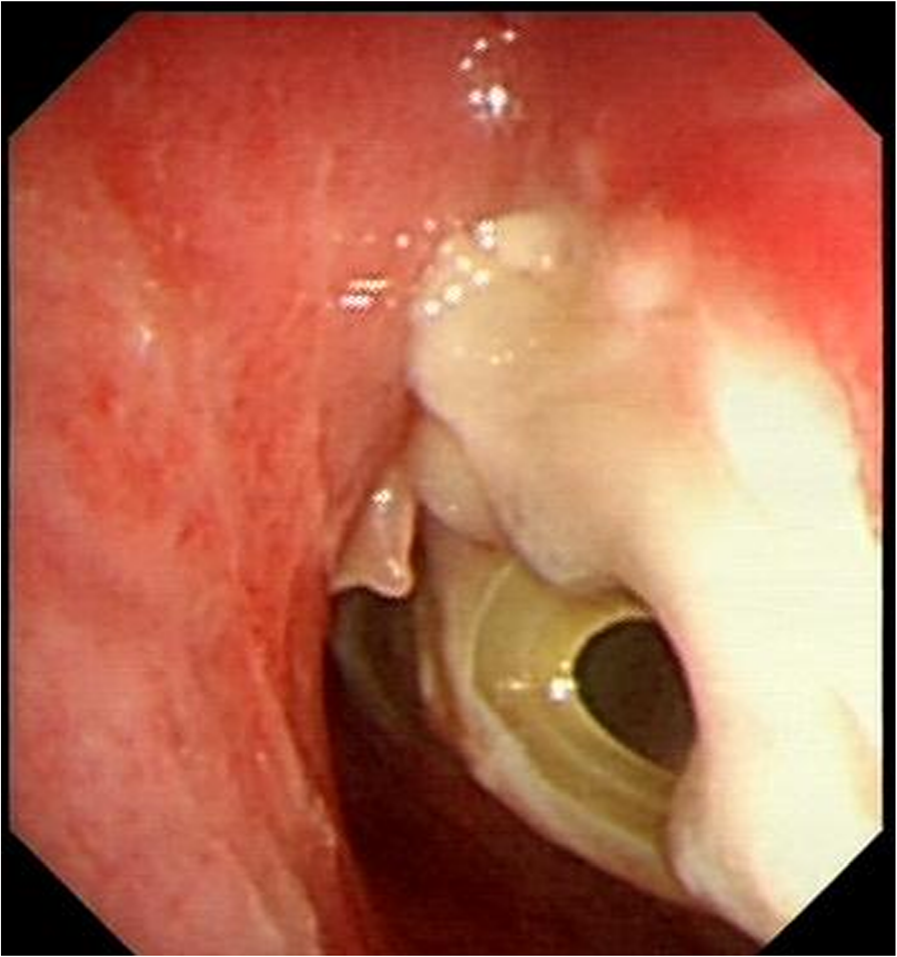
After biopsy was harvested, the end of mediastinal drainage tube emerged and located in the center of necrotic materials. Distal intermediate bronchus was compressed

**Fig. 4:**
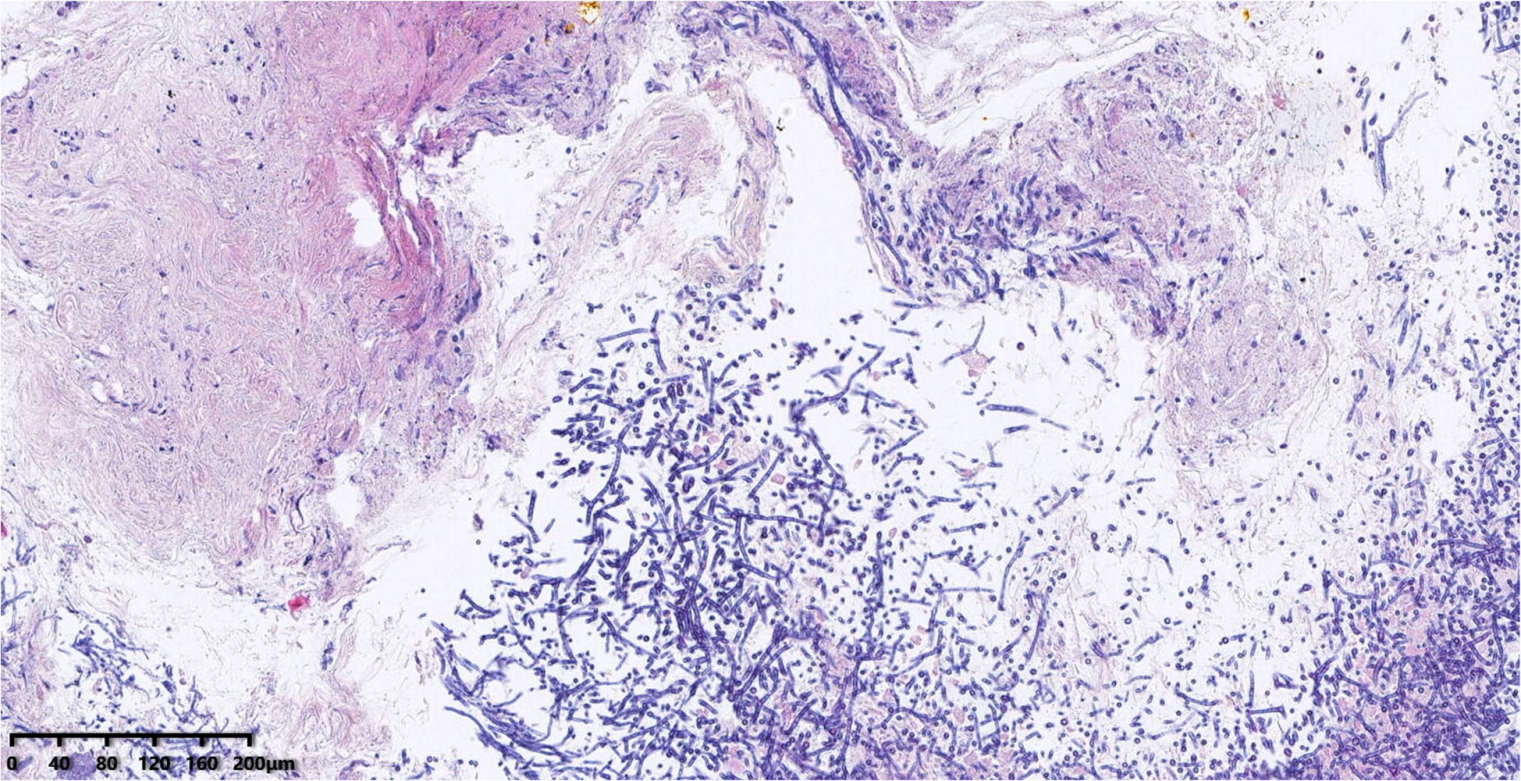
Micrograph of numerous fungal hyphae (H&E × 20)

A 50-year-old male patient with type 2 diabetes was observed air filling in mediastinal drainage tube, 12 days after esophagectomy for esophageal squamous carcinoma. Chest computed tomography presented normal anastomosis. From the lung window of chest multi-slice CT, the connection of intermediate bronchus and mediastinal drainage tube tract was observed (Fig. 1). Bronchoscopy revealed massive white necrotic materials attached the wall of intermediate bronchus. Distal intermediate bronchus was compressed (Fig. 2). After biopsy was harvested, the end of mediastinal drainage tube emerged and located in the center of necrotic materials (Fig. 3). Biopsy result is depicting numerous fungal hyphae (Fig. 4). Benign Intermediate bronchial fistula and fungal infection were diagnosed.

**Fig. 5:**
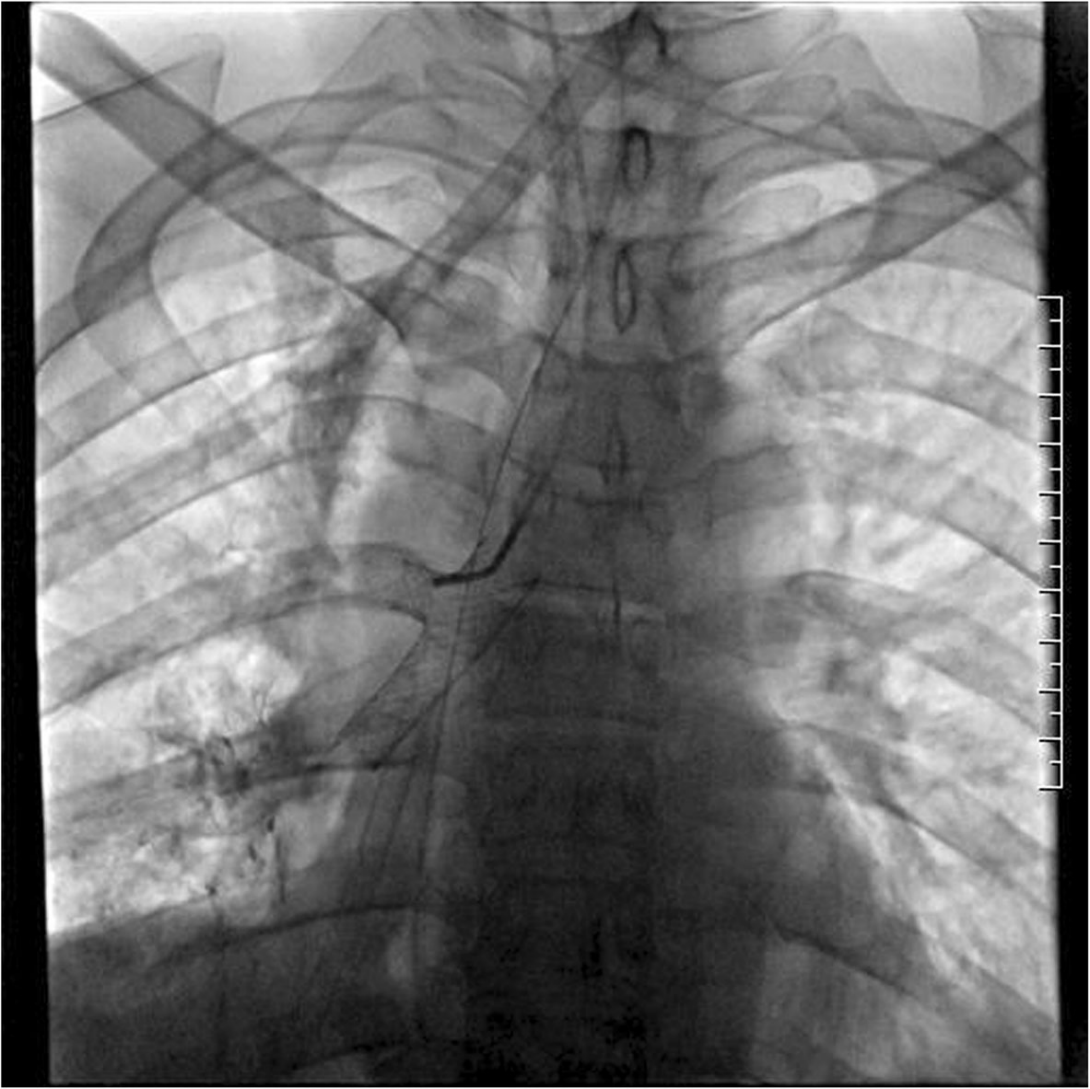
The stent was placed under the guidance of fluoroscopy

**Fig. 6:**
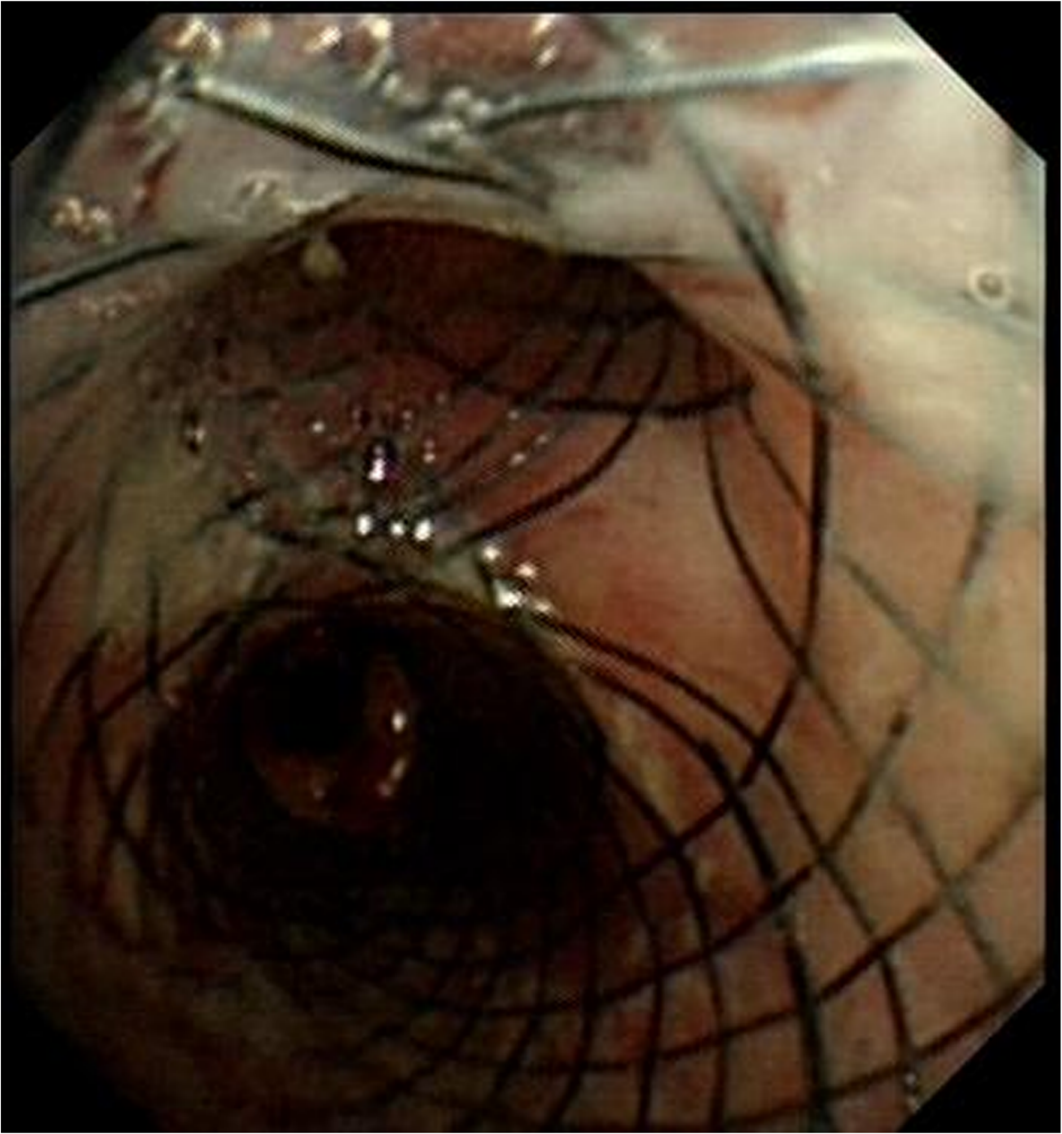
Bronchoscopy demonstrate the closure of fistula

**Fig. 7:**
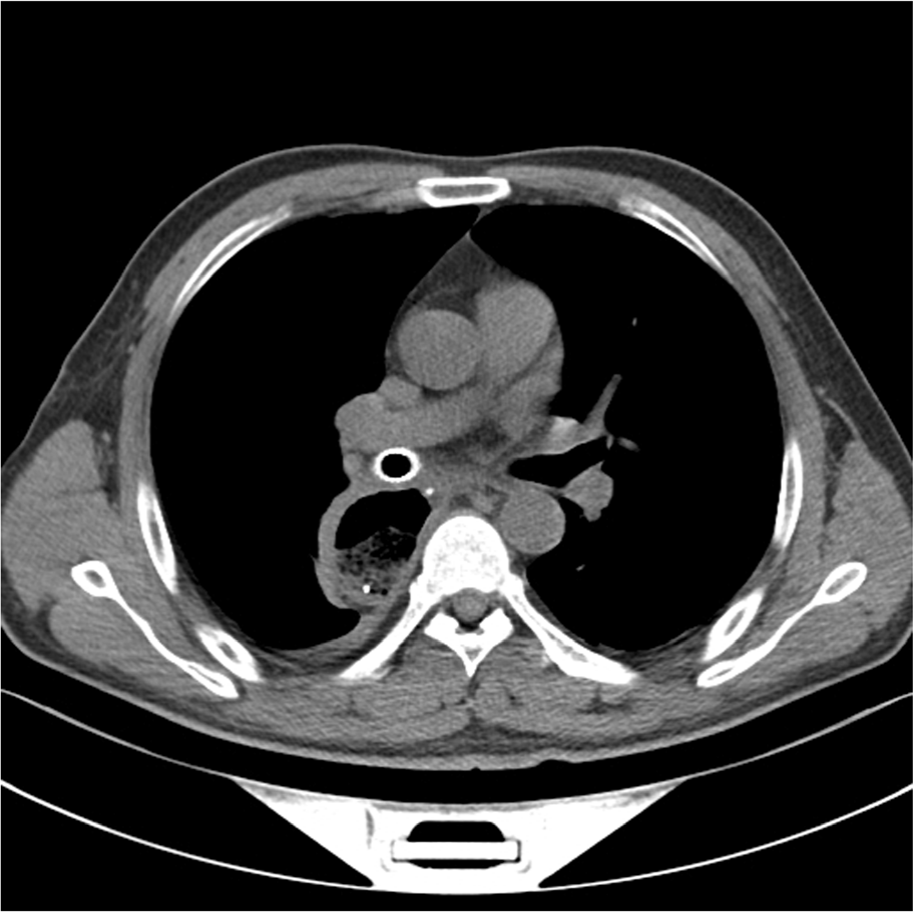
CT shows the fistula tract was cured

**Fig. 8:**
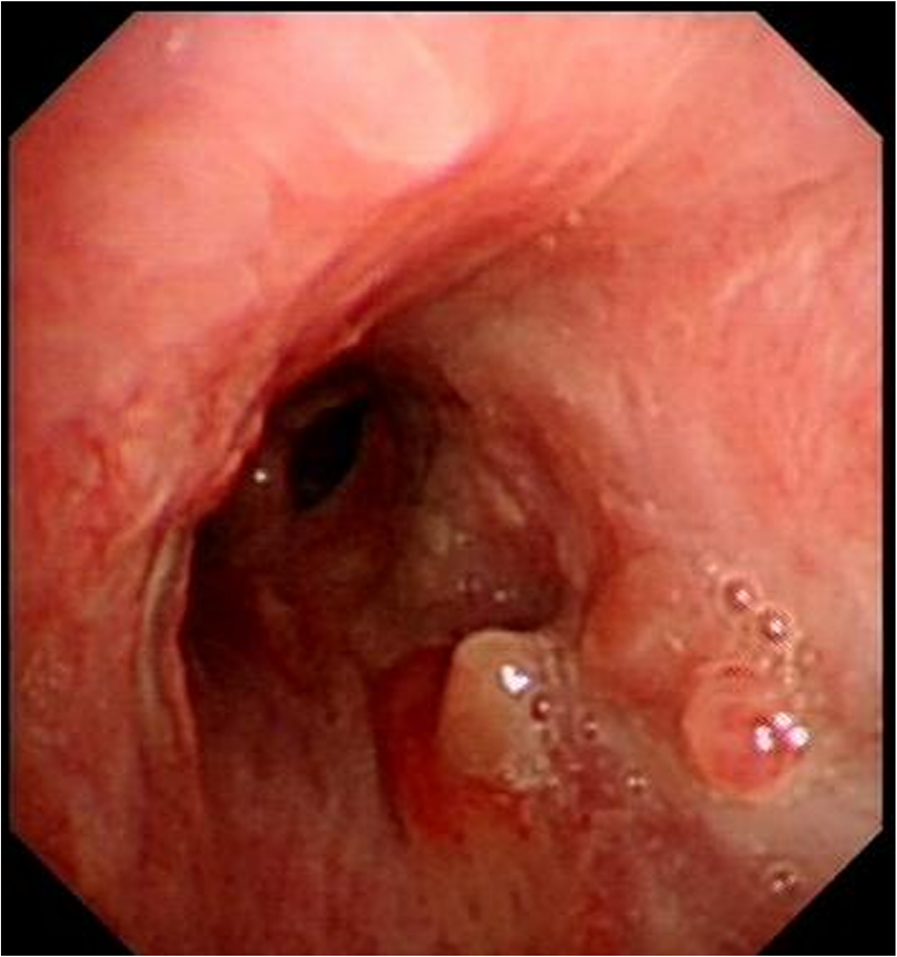
After stent remove, fistula was healed with some granulation hyperplasia

Silicon stent was the first choice for benign tracheobronchial fistula. However, small diameter Y-shaped silicon stent placement in second carina is not available in our hospital. An individualized Y-shaped covered self-expandable metallic stent (SEMS) temporary placement and antifungal treatment were developed. Initially, stent was designed depending on the diameter and length measurement result of right main bronchus, right upper bronchus and intermediate bronchus from CT. The diameter of bronchial part, upper lobe part and intermediate bronchial part is 14 mm,10 mm and 12 mm. The length is 10 mm, 10 mm and 25 mm, respectively. Under the guidance of fluoroscopy, two super-stiff guide wires were exchanged into the right lower and right upper lobe bronchi. Once the delivery system that contained Y-shaped covered SEMS in place, the stent was released (Fig. 5). No procedure related complications happened. Bronchoscopy were performed to demonstrate the closure of fistula (Fig. 6). Mediastinal drainage tube was withdrawn gradually. Forty days later, CT shows the fistula tract was cured (Fig. 7). The stent was successfully removed. Bronchoscopy revealed the fistula was healed with some granulation hyperplasia (Fig. 8). He was free from respiratory symptom during 1 year follow-up.

## Discussion and conclusions

Intermediate bronchial fistula formation caused by mediastinal drainage tube compression and fungal infection is rare. The major risk factor for fungal infection is immunodeficiency including transplantation, AIDS, prolonged therapy with corticosteroids and malignancy et.al. Uncontrolled diabetes mellitus could results mildly immunosuppression and increases the risk of bacterial infection. In our case, the combination of mediastinal drainage tube compression and destroy effect of fungal infection contribute to bronchial fistula formation.

Imaging findings are crucial for the diagnosis. From the lung window of chest multi-slice CT, the connection of intermediate bronchus and mediastinal drainage tube tract was observed. The specific finding of fungal infection is pseudomembrane. Fungal infection would bring completely demolished mucosa, transmural bronchial necrosis and pseudomembranes. The biopsy results depicting numerous fungal hyphae. Based on the evidence above, the diagnosis of benign intermediate bronchial fistula was made.

Early diagnosis and antifungal treatment may significantly improve the outcome. Arguder and colleagues reported a case of pseudomembranous aspergillus tracheobronchities, which caused tracheal perforation [1]. The patient was unsuitable for any invasive procedure because of very large perforation and died of respiratory failure. In our case, due to the fistula in intermediate bronchus, the lung function is not affect obviously. After the diagnosis of fungal infection from pathology was made, antifungal drug was given.

The treatment strategies of benign tracheobronchial fistula including surgery and endobronchial stenting. Considering the general condition of our patient, endobronchial stenting as a minimally invasive was developed. Small diameter silicon stent placement was the first choice in our case, but it is not available in our hospital, only metallic stent can be used. Although FDA warning uncovered metallic stenting in benign tracheal stenosis due to granulation hyperplasia. The application of covered metallic stent benefit many patients with tracheal fistula and stenosis [2–4]. The obstacles of the early years of metallic stent implantation involved the limitations of stent sizes. Ill-matched stent including undersized stent and oversized stent may cause undue friction and pressure, result in excessive granulation tissue formation [5]. To minimize the complications of granulation tissue formation after metallic stent placement, we ordered an individualized covered metallic stent to match the diameter of bronchus. Post-stent 40 days, sputum retention was observed through bronchoscopy, no stent migration and severe granulation hyperplasia happened.

Thus, we conclude that: 1. Tracheobronchial fistula caused by the combination of mediastinal drainage tube compression and fungal infection is rare. It is characterized by pseudomembranous formation on the wall of airway. 2. Uncontrolled diabetes mellitus could results mildly immunosuppression and increases the risk of bacterial infection. 3. Timely stenting could boost the healing of fistula via granulation tissue proliferation.

## Data Availability

Please contact author for data requests.

## Abbreviations

CT: Computed tompgraphy
SEMS: Self-expandable metallic stent
